# Contrast-Enhanced Ultrasound Follow-Up for Acute Pyelonephritis Patients

**DOI:** 10.3390/healthcare11212899

**Published:** 2023-11-03

**Authors:** Andrea Boccatonda, Stefano Venerato, Damiano D’Ardes, Giulio Cocco, Cosima Schiavone, Susanna Vicari

**Affiliations:** 1 Internal Medicine, Bentivoglio Hospital, AUSL Bologna, 40010 Bentivoglio, Italy; stefano.venerato@ausl.bologna.it (S.V.); susanna.vicari@ausl.bologna.it (S.V.); 2 Department of Medicine and Aging Science, Institute of “Clinica Medica”, “G. d’Annunzio” University of Chieti, 66100 Chieti, Italy; damiano.med@libero.it; 3 Internistic Ultrasound Unit, SS Annunziata Hospital, “G. D’Annunzio” University, 66100 Chieti, Italy; cocco.giulio@gmail.com (G.C.); cschiavone@unich.it (C.S.)

**Keywords:** kidney, CEUS, ultrasound, pyelonephritis, infection

## Abstract

Contrast-enhanced ultrasound (CEUS) is increasingly used in clinical practice as the first diagnostic method in patients with suspected pyelonephritis rather than abdominal CT with contrast medium, especially in young subjects. We performed a retrospective analysis on patients in for whom a CEUS examination was utilized as a follow-up method after acute pyelonephritis as normal clinical practice. Through evaluating all patients, in terms duration between CEUS examination and normalization (healing) of the renal disease, we found that the mean duration is 25.9 days. Our ultrasound findings did not induce any therapeutic modifications, not even in the cases in which the examination was repeated several times. Therefore, setting up a CEUS follow-up examination after 25 days from the first diagnosis can reduce the number of repeated tests, benefitting patients and the healthcare system in terms of reducing costs.

## 1. Introduction

Urinary tract infection (UTI) refers to several clinical phenotypes, such as cystitis, pyelonephritis, prostatitis, urosepsis, and catheter-associated UTI [1]. UTI is the most frequent urologic problem in the world, affecting about 150 million patients/year [2]. UTI is considered the most relevant nosocomial infections (31%) in American intensive care units, and it is the second most common nosocomial infection in Europe [2]. UTI is related to several micro-organisms, such as Gram-positive bacteria, Gram-negative bacteria, and fungi. Uropathogenic *Escherichia coli* is the commonest bacterium correlated with both complicated and uncomplicated UTI [3]. Acute pyelonephritis is an acute infection of the renal parenchyma and collecting system. It is related to ascending infection from the bladder or by hematogenous spread. Young sexually active women are the most affected subjects, with 90% of pyelonephritis induced by *Escherichia coli*. Low-virulent *Escherichia coli*, other Gram-negative and Gram-positive bacilli, and candida are responsible for pyelonephritis in adult men, elderly women, patients with urological problems, and hospitalized patients [4]. Fever, pain, nausea, vomiting, a burning sensation during urination, and increased urination frequency and urgency are the most frequent symptoms [4]. Complications are related to the obstruction of the urinary tract and inadequate clinical response to intravenous antibiotic treatment after 72 h. 

In the context of treating patients with UTIs, both magnetic resonance (MRI) and computed tomography (CT) provide high image quality. CT is often more readily available than MRI; however, both modalities can evaluate the renal parenchyma, the surrounding structures, and the urothelial wall [5]. Nowadays, CT is considered the gold standard imaging method for UTI evaluation due to its speed of execution, the fact that it shows anatomical and physiological details, and the fact that it can detect both renal and extra-renal diseases. MRI is required as a problem-solving method when CT is not conclusive. MRI is the imaging method of choice in pregnant women and in subjects with contrast medium contraindication. It is possible to distinguish two different pyelonephritis: diffuse and focal. If the infection was spread through blood, it is possible to detect round peripheral parenchymal lesions with hypoenhancement; ascending infection could also present with wedge shaped areas of hypoperfusion [6]. The diffuse type is characterized by renal enlargement, thickened fascia, and poor enhancement of the parenchyma [7].

Contrast-enhanced ultrasound (CEUS) is increasingly used in clinical practice as the first diagnostic method used for patients with suspected pyelonephritis rather than abdominal CT with contrast medium, especially in young subjects [8,9]. CEUS can show areas of hypoenhancement in the arterial phase and early de-enhancement in the late phase [10,11]. A previous work demonstrated the same diagnostic accuracy between CEUS and CT, especially for focal lesion detection [12]. According to 2017 EFSUMB guideline, CEUS is indicated to evaluate renal abscess in complicated acute pyelonephritis [13].

There are few data in the literature on the role of CEUS as a follow-up imaging method after the acute phase of the disease. The main aim of our study was to evaluate the role of CEUS as a follow-up method for pyelonephritis.

## 2. Materials and Methods

We performed a retrospective analysis on patients for whom a CEUS examination was utilized as a follow-up method after acute pyelonephritis as normal clinical practice in our internal medicine ultrasound unit. Due to the descriptive nature of the study, the timing of the follow-up was set by each physician who managed the patient during hospitalization and discharged the patient. Data regarding basic characteristics; laboratory test values, including C-reactive protein (CRP); procalcitonin; and blood count at admission and on discharge were collected. The results of urine cultures and any antibiograms were also evaluated. Informed consent was obtained from all patients enrolled in the study. This study is a descriptive retrospective study. The local Research Ethics Committee confirmed that no ethical approval was required.

### 2.1. Ultrasound and CEUS Technique

Ultrasound examinations were performed via convex probe (Philips, Eindhoven, Netherlands, Affiniti 70). The kidneys were studied by using axial and longitudinal scans. Our B-mode ultrasounds were focused on detecting hypoechoic lesions related to pyelonephritis, the presence of hydronephrosis, and observing the presence of echo-structural alterations such as perirenal fluid collections. The study was completed via duplex ultrasound evaluation.

CEUS examinations were performed using the C5-1 convex probe (Philips) with a frequency range of 1.0–5.0 MHz; the dynamic range was set to 42, and the mechanical index was low. 

The CEUS examination was performed according to the methods recognized by the relevant guidelines [13,14]. The diameter of the venous line was 20 gauge or larger to minimize the destruction of microbubbles as they passed through the cannula, with the length being kept as short as possible. 

An ultrasound contrast agent (UCA) (SonoVue™, Bracco, Milan, Italy), which acted as a pure intravascular agent consisting of micro-bubbles (1–7 micron) that contain sulfur hexafuoride encapsulated by a phospholipid shell, was employed.

The injection bolus for SonoVue™ was delivered at approximately 1–2 mL/s. Immediately following the injection of the contrast medium, a bolus of (5-) 10 mL of saline was administered to flush the line at approximately 2 mL/s. The recommended dose of SonoVue™ for the detection and characterization of kidney lesions was 2.4 mL (1/2 vial) [13,14]. 

**Figure 1:**
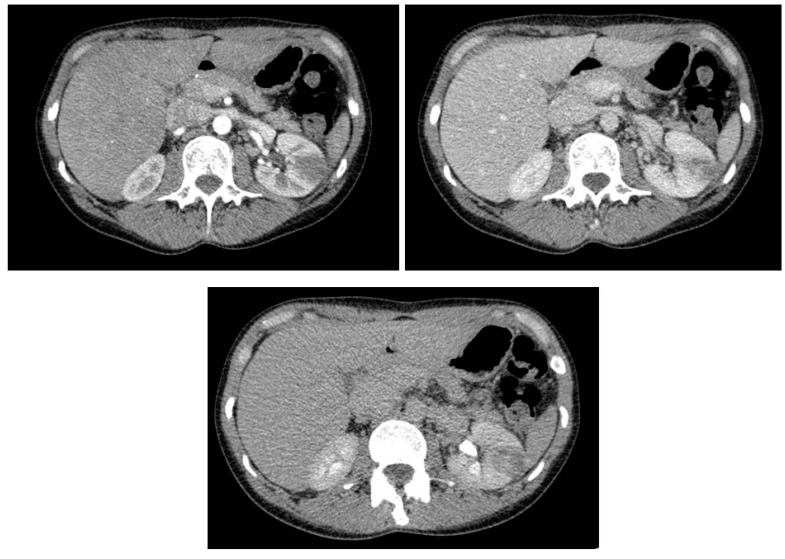
Abdomen CT-scan with contrast medium. The left kidney is slightly larger in size than the contralateral one, and it presents a large cortical area of reduced post-contrast impregnation in the middle with a maximum axial diameter of approximately 2.8 cm. The findings are referable to acute pyelonephritis.

**Figure 2:**
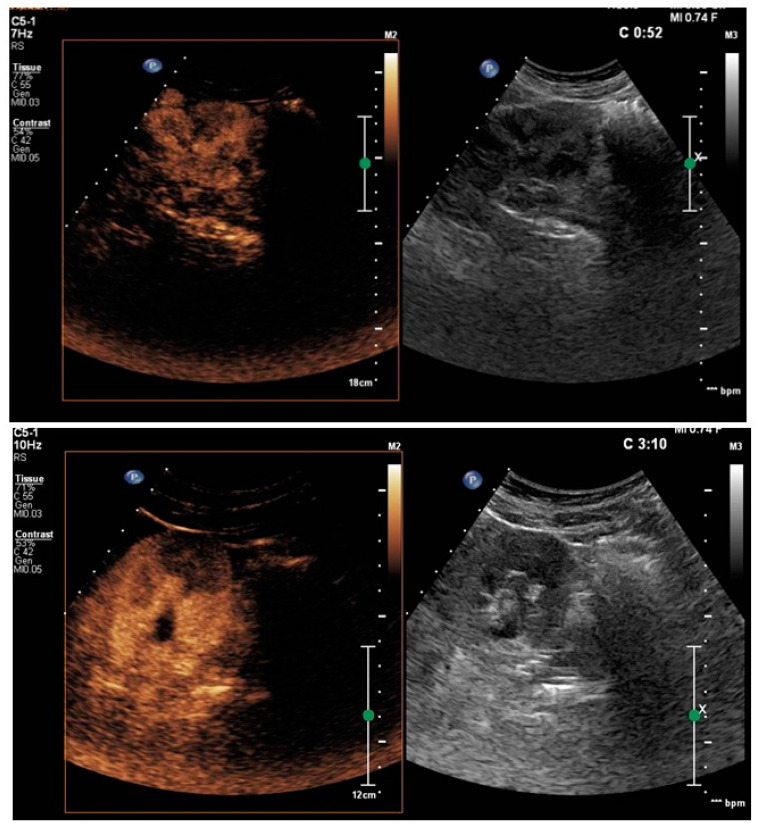
First CEUS follow-up (13 days after the first diagnosis). In the middle of the left kidney, an oval area of altered perfusion of approximately 27 mm persists, compatible with the outcome of pyelonephritis.

**Figure 3:**
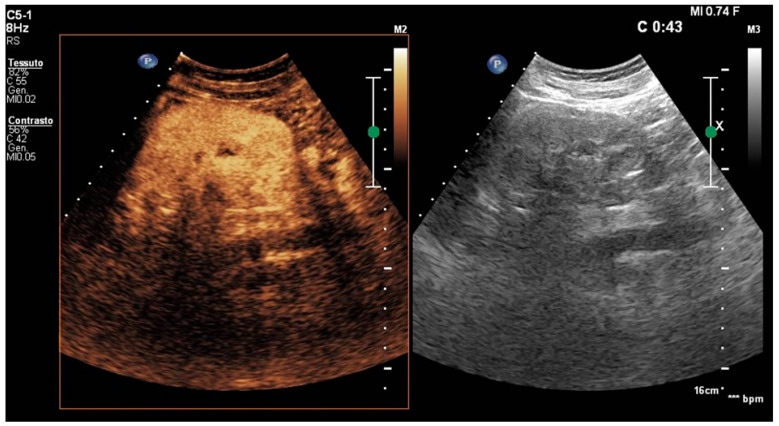
Second CEUS follow-up (30 days after the first diagnosis). There is a complete resolution of the pyelonephritis on the left kidney, with regular contrast taking over the cortical portion.

Pyelonephritic lesions were detected as wedge-shaped or as round hypoechoic lesions in the cortex and/or in the medullar region. The late parenchymal phase favors the detection of those lesions because the findings are more variable in the other phases. Most of the lesions are initially hypoechoic before turning isoechoic and back to hypoechoic again. Focal pyelonephritis with small abscess appears as focal pyelonephritis with areas of non-enhancement, related to small abscesses, inside (Figure 1, Figure 2 and Figure 3).

### 2.2. Statistical Analysis

All of the variables were tested to verify the distribution; variables with no normal distribution were analyzed using non-parametric tests. Pearson coefficients and Spearman’s rho coefficients were analyzed, based on the cases, for bivariate analyses. A *p* value of less than 0.05 was considered statistically significant. All the tests were two-tailed variables, and the analyses were carried out using SPSS (VERS. 22.0, Chicago, IL, USA).

## 3. Results

Twenty-eight CEUS examinations focused on the kidney were performed as a follow-up measure after a diagnosis of acute pyelonephritis in 23 patients (Table 1); in five cases (17.8%), the CEUS was repeated several times. B-mode ultrasound detected nephrolithiasis in four patients (17.3%) and hydronephrosis in six cases (26%). Urine culture was positive for *Escherichia coli* infection in seven cases (30.4%) and Klebsiella pneumoniae infection in two patients (8.6%). In our sample, there were no cases of pyelonephritis on transplanted kidneys or in pregnant women.

The mean age of all patients was 49.2 ± 21.4 years (mean age of males: 69 years; mean age of females: 43.5 years) (Table 2). The patients were re-evaluated at a mean of 23.7 ± 18.6 days (range 7–81 days) after the first diagnosis. Subjects > 40 years were reevaluated at a mean of 33.7 days; subjects aged < 40 years were re-evaluated at a mean of 15.1 days. With reference to the five cases that required multiple follow-ups, they were all female, with a mean age of 29 years (range 21–39 years). The mean timing of the first follow-up examination was found to be 11.8 days (range 8–14 days). Considering that one patient was lost to the next follow-up, in the remaining four subjects, mean time to normalization of renal damage was 42.5 days (range 25–71 days). Considering all 22 patients, mean time to normalization was 25.9 ± 19.3 days. 

Days of resolution were unrelated to white blood cell count (WBC) at admission (*p* = 0.272), C-reactive protein (CRP) at admission (*p* = 0.509), and procalcitonin (PCT) at admission (*p* = 0.978), as well to WBC at discharge (*p* = 0.690), CRP at discharge (*p* = 0.136), and PCT at discharge (*p* = 0.635). Notably, the ultrasound findings did not show a therapeutic variation in terms of resumption or change of antibiotic therapy for any patient.

## 4. Discussion

Ultrasound and CEUS can play a relevant role in the diagnosis and follow-up of pyelonephritis. Based on the distribution of renal parenchymal lesions, acute pyelonephritis can be divided into focal, multifocal, or diffuse. In our sample, 82.6% of cases were monoliteral, and 34.7% were multifocal. 

Pyelonephritis is characterized by abundant inflammatory cells in the interstitium and tubules [15]. Relevant capillary damage can be presented, along with the presence of intraluminal leukocyte and fibrin plugs besides the inflammatory infiltrate and edema [16]. Those pathological changes are responsible for the hypoechoic feature of the lesions on CEUS and the lower enhancement with respect to the surrounding cortex. When there are focal areas of pyelonephritis, they appear more evident during the late phase because they are more hypoechoic in that phase. Notably, CEUS can evaluate renal parenchymal perfusion to detect complications of acute pyelonephritis, such as abscesses [17].

Previous studies have reported that CEUS has a lower diagnostic accuracy than CT in the context of the diffuse form of acute pyelonephritis due to the difficulties in having to simultaneously image the other kidney. Anyway, the diagnostic accuracy of CEUS has been estimated to be 100% as a positive predictive value and 89% as a negative predictive value in the evaluation of focal and multifocal pyelonephritis [12]. 

In our study, CEUS was the first and only imaging technique in 35.7% of cases; CT was performed on 50% of patients. Both the diagnostic methods were employed in 14.2% of cases. No case required the use of magnetic resonance imaging.

Currently, the follow-up of acute pyelonephritis is not yet well standardized. Several guidelines recommend antibiotic therapy for 7 to 10 days in adults [18,19]. For trimethoprim, a 14-day course was recommended because there was no evidence for course lengths shorter than 14 days [18,19]. A recent work showed how patients who were appropriately treated by empirical antibiotics had shorter hospital stays (8 vs. 10 days, *p* = 0.001) and lower medical costs than those who were inappropriately treated [20].

To the best of our knowledge, this is the first work to evaluate data on the use of CEUS in the follow-up of pyelonephritis in adults. At our hospital, several follow-up CEUS examinations are usually performed in cases of pyelonephritis, with each taking place at different times depending on clinical judgment at the time of discharge (mean 23.7 days). We observed that the mean time of CEUS follow-up was significantly lower in patients under the age of 40 years than in the over 40s (15.1 vs. 33.7 days). We hypothesize that this finding can be explained by the belief among clinicians that a more rapid recovery is likelier in young subjects compared to elderly subjects. In the five cases in which more than one ultrasound assessment was required for normalization, all patients were <40 years, with a mean age of 29 years. By analyzing the five cases, they withdrew antibiotic treatment at the time of ultrasound follow-up; the antibiotic treatment was carried out for a week, and in two cases, it was targeted due to the finding of positive urine cultures following an antibiogram (*E. coli* treated with piperacillin/tazobactam). The number of cases is too small to hypothesize that a specific bacterium or antibiotic regimen may be responsible for different recovery times.

One of the most relevant data points is that there is a mean time of 25.9 days to observe the normalization of renal disease via CEUS by evaluating all patients. Therefore, data relating to the five cases in which the second CEUS examination had to be repeated were probably due to the fact that ultrasound follow-up was set with too short a time frame (mean 11.8 days).

In a previous work, a series of follow-up CEUS exams were performed in seven children who were diagnosed with renal abscesses [21]. Renal parenchyma scars, thickening of kidney cortex, or defects of perfusion corresponding to the abscess were not detected in follow-up exams after completing a treatment regimen [21]. 

Our ultrasound findings did not indicate any therapeutic modifications, not even in the cases in which the examination was repeated several times. 

No adverse reactions among our patients were recorded following the administration of the ultrasound contrast medium. The present study excluded pregnant women and transplant patients. By analyzing studies in the literature and the technical data sheet of the contrast medium, we found that performing a CEUS examination on pregnant women is not currently recommended. For patients with renal transplants, there are no data in the literature reporting contraindications to CEUS examination; furthermore, it is recommended in patients who cannot perform contrast medium on CT examinations due to renal failure.

## 5. Study Limitations

Our study has several limitations, the most obvious of which is the descriptive and retrospective nature of the study. Moreover, the small number of cases considered in this study could limit the statistical power and generalizability of our results. There was no standard protocol to determine the optimal timing of CEUS follow-up, thus making the results less reliable, although this finding may reflect real-world data. 

Other limitations include interoperator variability and the fact that the operators did not perform the examination in a blinded manner. There was no control group in this study, but including one could have helped to clarify that there are no clear recommendations on the follow-up methods for patients after the acute phase of pyelonephritis, as there is no gold standard reference method. The results of the present study represent descriptive data that could be very useful for planning prospective controlled studies aimed at verifying clinical utility, even in the long term (e.g., 3–6 months).

## 6. Conclusions

CEUS follow-up after the acute phase of pyelonephritis does not present substantial advantages from a strictly clinical point of view, and it does not induce therapeutical changes. The request for a CEUS examination on follow-up is therefore based only on the desire to verify the complete healing by the imaging method used. Considering the limitations of the present study, including the retrospective and descriptive nature of the study, the data derived from our study suggest a mean healing time of 25 days for pyelonephritic lesions. Therefore, setting up a CEUS follow-up examination 25 days from the first diagnosis could help to reduce the number of repeated tests, benefiting patients and the healthcare system in terms of reducing costs. Regarding future studies, a multi-center study would improve the external validity of these findings.

## Figures and Tables

**Table 1:** Characteristics of patients included in the study.

Clinical Characteristics	Value
Age (years)	49.2 ± 21.4
Female sex	17 (73.9%)
Ultrasound follow-up (days)	23.7 ± 18.6
Pyelonephritis resolution (days)	25.9 ± 19.3
WBC admission (×10^9^/L) [3.60–10.50]	11.3 ± 3.3
CRP admission (mg/dL) [<0.50]	15.6 ± 7.3
PCT admission (ng/mL) [<0.50]	6.4 ± 5.5
WBC discharge (×10^9^/L) [3.60–10.50]	6.7 ± 2.1
CRP discharge (mg/dL) [<0.50]	3.0 ± 2.3
PCT discharge (ng/mL) [<0.50]	0.7 ± 0.4

Continuous variables are expressed as mean and standard deviation; the discrete variables are expressed as number and percentage. The measure units are presented in round brackets; the reference values are presented in square brackets. Abbreviations: WBC, white blood cell count; CRP, C-reactive protein; PCT, procalcitonin.

**Table 2:** Characteristics of pyelonephritis.

Characteristics of Pyelonephritis	*n* (%)
Monolateral	19 (82.6%)
Monofocal	14 (60.8%)
Multifocal	8 (34.7%)
Bilateral	4 (17.3%)
Nephrolithiasis	4 (17.3%)
Hydronephrosis	6 (26%)
*Escherichia coli* + urine culture	7 (30.4%)
Klebsiella pneumoniae + urine culture	2 (8.6%)
First diagnosis by CEUS	10 (35.7%)
First diagnosis by CT	14 (50%)
First diagnosis by CEUS + CT	4 (14.2%)

The discrete variables are expressed as number and percentage. Abbreviations: CEUS, contrast-enhanced ultrasound; CT, computed tomography.

## Data Availability

Data are available from corresponding author upon request.
